# Drawing a pandemic vulnerabilities' map: The SoNAR-global Vulnerabilities Assessment digital and its output

**DOI:** 10.3389/fsoc.2023.1127647

**Published:** 2023-02-08

**Authors:** Concetta Vaccaro, Francesca Romana Lenzi, Gabriella Addonisio, Daniele Gianfrilli, Anna Maria Volkmann, David Napier, Tamara Giles-Vernick

**Affiliations:** ^1^Health and Welfare Unit, Censis Foundation, Rome, Italy; ^2^Laboratory of Psychology and Social Processes in Sport, University of Rome “Foro Italico”, Rome, Italy; ^3^Department of Experimental Medicine of the Sapienza University of Rome, Rome, Italy; ^4^Department of Anthropology, Science, Medicine, and Society Network, University College London, London, United Kingdom; ^5^Anthropology and Ecology of Disease Emergence Unit, Institut Pasteur, Paris, France

**Keywords:** COVID-19, qualitative methodology, social vulnerabilities, urban health, digital methods, social determinants of health

## Abstract

This paper describes the process, advantages and limitations of a qualitative methodology for defining and analyzing vulnerabilities during the COVID-19 pandemic. Implemented in Italy in two sites (Rome and outside Rome, in some small-medium sized municipalities in Latium) in 2021, this investigation employed a mixed digital research tool that was also used simultaneously in four other European countries. Its digital nature encompasses both processes of data collection. Among the most salient is that the pandemic catalyzed new vulnerabilities in addition to exacerbating old ones, particularly economic. Many of the vulnerabilities detected, in fact, are linked to previous situations, such as the uncertainties of labor markets, having in COVID-19 to the greatest negative effects on the most precarious workers (non-regular, part-time, and seasonal). The consequences of the pandemic are also reflected in other forms of vulnerability that appear less obvious, having exacerbated social isolation, not only out of fear of contagion, but because of the psychological challenges posed by containment measures themselves. These measures created not mere discomfort, but behavioral changes characterized by anxiety, fearfulness, and disorientation. More generally, this investigation reveals the strong influence of social determinants throughout the COVID-19 pandemic, creating new forms of vulnerability, as the effects of social, economic, and biological risk factors were compounded, in particular, among already marginalized populations.

## 1. Introduction

In 2021, the European funded social sciences network for preparedness and response to epidemics, SoNAR-Global, undertook a five country, qualitative investigation of social, economic and health vulnerabilities as a consequence of the COVID-19 pandemic. The goal of this new initiative was to examine the complex, interacting factors that shape COVID-19 vulnerabilities in Italy, France, Germany, Malta, and Slovenia. In addition to characterizing COVID-19 vulnerability and resilience, this newly funded study highlighted the need for community involvement, and the potential role of the social sciences in strengthening public health responses to epidemics (https://www.sonar-global.eu/).

The Censis Foundation and Sapienza University of Rome conducted the Vulnerability Assessment among 200 individuals in Rome and outside Rome, in small towns in Lazio. We report here both the findings of this Assessment which then formed the basis for the Community Engagement activity and their co-translation with multiple stakeholders into new strategies for allocating public response resources more effectively and efficiently.

This article reports on the activities and results of the Vulnerability Assessment conducted in Italy as part of the SoNAR-Global project, also due to its interest in the use of digital methods. The results of the Community Engagement will be the subject of a further publication.

The concepts of Vulnerability and Community Engagement are particularly amenable to a systematic social sciences analysis of the impacts of infectious diseases on lived experiences in real world contexts, because they address individual, social and contextual factors shaping exclusion and resilience (Kaufman et al., 2003; Jeleff et al., 2019). As reported in a recent literature review, some historical and structural factors (i.e., history of marginalization, institutionalized racism and discrimination, trust in the public policy) and other aspects of vulnerability are important elements of the challenges faced by communities.

The concepts of Vulnerability Assessment and of Community Engagement are considered by a large area of study, yet lack clearly agreed upon definitions, and uses of the terms vary across disciplines, even if they recently benefit from an increasing interest in social science, public health disciplines, research programs, government bodies, and NGOs projects (Osborne et al., 2021a). They are broadly related to the recognition of the social, cultural and political nature of the diseases: the power of a disease is rooted by a number of aspects not directly related to the medical dimension itself and explain the generation of forms of vulnerabilities that impact on a general community risk (Janes et al., 2012; Stellmach et al., 2018). While the concept of vulnerability includes aspects can contain a multitude of factors that impact on ill health, Community Engagement can be defined as a related practice that “seeks to utilize social networks to mitigate threats to infectious diseases” (Osborne et al., 2021a). The practice of Community Engagement aims to reach those groups that experience disease more than others (Southall et al., 2017). This is a reason why a “successful Community Engagement may therefore address those vulnerabilities associated with differing historical, social, political, and economic worldviews and situations” (Osborne et al., 2021a).

Community Engagement has been defined by Brunton et al. (2017), who outline concepts related within Community Engagement as a strategy adapted to each unique community in an utilitarian term, if it is used to boost participation in or agreement with an intervention, or as social justice if it helps to focus on inequalities. This identification with a strategy is shared with a large number of international projects and practices, such the WHO'S risk communication and community engagement (RCCE) strategy, which underline the importance of including socio-behavioral analyses for Community Engagement efforts (WHO, 2021).

Few clear and comprehensive guidelines for Community Engagement exist on the base of different perspectives and actors involved in the process that help understanding what constitutes Community Engagement. UNICEF proposes a set of minimum quality standards and indicators that answers the demand for a cohesive approach to Community Engagement in the context of global public health priorities, proposing a clear definition of Community Engagement followed by four domains (core Community Engagement standards, standard supporting implementation, standards supporting coordination and integration and standards supporting resource mobilization) and 16 areas for understanding Community Engagement in practice (UNICEF et al., 2020, p. 12).

On the other side, the concept of vulnerability can be analyzed from its uses in the study of infectious disease. It is composed by an individual (biological) vulnerability and by a vulnerability of systems or structures (Ezard, 2001). Such as resilience, vulnerability is not fixed state, but it occur for various reasons and a number of them can be redirected to social determinants of health (Wilkinson and Marmot, 2003; Marmot and Wilkinson, 2005; Marmot et al., 2008; Wilkinson and Pickett, 2010; World Health Organization, 2013; Marmot, 2014, 2015; Wilkinson et al., 2017; Napier and Volkmann, 2023). One of the study already concluded on the same topic as this in the one of Linder et al. (2018) on urban diabetes, as a part of the Cities Changing Diabetes program (https://www.citieschangingdiabetes.com), on which other studies have been run in Rome and Milan (Italy) (Nicolucci et al., 2019). Linder et al.'s study identify social and cultural aspects of vulnerability and how they differ across different types of community members. They use the changes between groups to argue for responses that are adapted to “composite vulnerability, that is, vulnerability that encompasses social, neighborhood and individual-level attributes” (p. 2).

Measuring vulnerability or using it as a strategic concept within Community Engagement could offer theoretical paths from the social sciences to conquer a place in the research and practice of Community Engagement globally. On this we refer to *The Rapid Assessment of Vulnerable Populations: a 'barefoot' Manual* (Napier, 2014).

As this literature overview shows, studies examining vulnerability and resilience within their community contexts share important characteristics, especially in terms of assessing levels of local engagement, meaningful social participation, and agency and individual empowerment. In addition, establishing how these may be positively impacted by intersectoral collaboration is an additional benefit of the approach outlined herewith (Osborne et al., 2021b). The present study contribute to the mentioned literature adding an important dowel to the scenario of the use of social sciences to analyze the diffusion of diseases and the development of new complex forms of vulnerabilities. The present research bring some new perspective not only because social factors become central to analyze the impact of Infectious diseases during the pandemic course, but also because the Community Engagement—although this last practice is not investigated by the paper—has been applied as the main strategy to assess vulnerabilities and to find solutions.

## 2. Materials and methods

The Vulnerability/Resilience Assessment, developed as a method by David Napier and Anna-Maria Volkmann of University College London, is a field-based tool for systematically collecting and analyzing heterogenous data relevant to the lived experiences of those made health vulnerable by a variety of compounding and often local risk factors. With this tool, data is collected on individual knowledge of and experiences with formal and informal aid and assistance. Alongside measuring standard vulnerability indicators (degree of isolation, capacity to manage health, trust in government, etc.), the tool identifies specific tipping points that limit or enhance health agency. Within the SoNAR-Global project framework, the Vulnerability/Resilience Assessment tool was adapted to address consequences of the COVID-19 pandemic (Napier, 2014; Ministero della Salute, 2020; Lenzi, 2021; COVID-19 Surveillance Group, 2022).

The first step consisted of carrying out 200 1–3 h closed- and open-question interviews. These were distributed across population categories suspected to be most adversely affected by the pandemic. The selection of the population and the number of interviews were selected on the bases of previous methodological steps of the SoNAR-Global and of other researchers conducted by the same group (Napier, 2014). The principle controlling the numbers of interviews derives from our years of Vulnerability/Resilience Assessment work in Cities Changing Diabetes project and other Vulnerability Assessment projects, which have demonstrated that we usually reach saturation for any “case definition” (being a set of shared risks working together in the lived experiences of our interviewees) once we have carried out no fewer than 10–15 detailed Vulnerability/Resilience Assessment for any given “case definition.” The team suggested some initial social or occupational categories whom we hypothesized had been adversely affected by the pandemic and its control measures, although we did not fix the number of interviews to be conducted for each category.

Our recruitment strategy was designed to build on these categories to identify hidden vulnerabilities has listed and collected in Table 1. Following the identification of initial participants, we used snowball sampling, requesting that initial participants put us in contact with others they considered to be as, or more, adversely affected by the pandemic than themselves. Though this type of sampling does not lead to statistically representative samples, it nonetheless provides an excellent exploratory technique, especially when trying to identify specific populations that traditional survey techniques fail to access.

**Table 1:** Respondents by type of initial vulnerability (absolute values and %).

	**a. v**.	**val.%**
Lonely people	31	16.3
Seniors over 70	15	7.9
Families with disabled or non-self-sufficient elderly/disabled/people with Down syndrome, etc.	9	4.7
People with chronic disease	50	26.3
Families with children up to 14 years/single-parents families	26	13.7
Students in DAD	21	11.1
People in precarious housing	13	6.8
Earthquake victims	7	3.7
Nurses	8	4.2
Caregivers (professional)	7	3.7
Caregivers (familiar)	10	5.3
Teachers	2	1.1
Deniers about COVID	8	4.2
People with psychological problems	8	4.2
Transgenders and non-binary	2	1.1
Women victims of violence	2	1.1
Families with financial difficulties	30	15.8
People with familiar trouble (in difficult divorce process)	6	3.2
People who have had COVID or long COVID	16	8.4
Smartworkers	24	12.6
Show business workers/dealers in penalized sectors	17	8.9
Unemployed	21	11.1
Precarious workers	42	22.1
Workers temporarily suspended from work with partial wages (*Cassa integrazione*)	13	6.8
Immigrants	12	6.3
Total	190	100.0

Seventeen trained interviewers carried out the 200 interviews (190 of which were usable), administering to each interviewee two questionnaires (a quantitative and a qualitative one) in Italian. We conducted these extended interviews in Lazio, where 100 interviews were carried out in Rome and 100 outside Rome, in small to medium-sized rural municipalities.

An initial, closed-question survey yielded demographic, socioeconomic and health data on each participant, whereas the detailed qualitative interview guide investigated three general thematic areas:

- the public and private Service Domain, in which detailed information is gathered regarding which services are used or not used, and the decision-making practices that impact knowledge and utilization patterns;
- the Community Domain, which focuses not only on social integration, but on the ability of communities, local advocacy groups, and particular stakeholders to mobilize, nourish, and harness social capital;
- and the Vulnerability Domain, which aims both to demonstrate how vulnerability is produced when the compounding of otherwise heterogeneous risk factors seriously limits health agency, and to characterize the ways in which, and the *ab nevus* through which health agency might be restored.

All interviews were recorded and transcribed. All participants provided written informed consent, in compliance with EU Regulation 2016/679. The ethics committee of the Policlinico Umberto I of the University La Sapienza of Rome approved the Italian study with App. 6340 on 26/05/2021.

One salient feature of our methodology concerns the online conduct of the interviews. In order to comply with anti-COVID measures, most interviews were carried out online, using platforms such as Zoom or Teams. This necessity entailed a new way of conducting the interviews that did not impede their execution or impoverish the results. Indeed, the online conduct proved effective in producing richly detailed narratives.

The data obtained were processed using SPSS (28 version) processing software to analyze the closed response questionnaire, and NVivo software (1.5.2 version), a computer assisted qualitative analysis software (CAQDAS—Computer Assisted Qualitative Data Analysis Software), which allowed for the coding and organization of information. From this coding we conducted thematic analysis, the data analysis process was substantiated by the research team's interpretive contribution, which involves exploring the data, modifying and integrating the initial codes, and identifying salient themes that are supported by codes.

Here again, the methodological aspect should be emphasized, since it was a method characterized by an integrated approach of analysis that allowed the schematization and understanding of the contents of the interviews through a logical ordering of the information obtained thanks to the software, which formed the basis for the researchers' interpretation.

## 3. Results

Thanks to the sampling method and the indications of interviewees, eight new vulnerability categories were found compared to those initially selected for sampling.

The following table shows the categories of vulnerability taken into consideration, which are the result of a mix between the initial ones and the subsequent integrations resulting from the sampling.

Table 2 reports the general characteristics of participants.

**Table 2:** General characteristics of the sample (absolute values and %).

	**a. v**.	**%**
**Age (from 18 to 91)**		
Upto 30	43	22.6
31–45	75	39.5
46–60	39	20.5
Over 60	33	17.4
**Gender**		
Women	99	52.1
Men	87	45.8
Other	4	2.1
**Location**		
Urban (Rome)	101	53.2
Rural area (other Latium municipalities)	89	46.8
**Modality for interview**		
In presence	75	39.5
Web/telephone	115	60.5
Total	190	100.0

One important finding is that consequences of the pandemic fell into two broad categories: those relating to the COVID-19 disease and those connected to pandemic control measures.

In fact, the second category of consequences is the result of political decisions that may have been wrong or excessive. Nevertheless, the effects of these different types of consequences are often linked: for example, the lockdown has not only impacted economic activity but increased fear for this unknown disease and also, reducing sociality, created anxiety and depression.

### 3.1. The consequences of the COVID-19 disease

COVID-19 disease not only affected those who fell ill, but also affected their carers, as well as the broader population, particularly in catalyzing generalized fear of an unknown disease, which in turn accentuated a sense of general vulnerability.

The fear of contagion and the fear of falling ill with COVID, given their health situation, and consequences of an unknown disease with potentially very serious outcomes certainly increased the vulnerability of chronic patients. Again, the most problematic situations concerned the frailest among the chronic patients, such as the elderly and the lonely elderly.

Caregivers have also experienced the fear of illness for the family members they care for and also for themselves, because of the possible consequences also with respect to their care commitment.

But there is also a very acute feeling of transversal vulnerability, fear and anxiety, linked to the characteristics of COVID, to the lack of knowledge and its unpredictability, which is independent of the presence of a previous situation of illness or fragility.

In addition to the distress created by the fear of illness, it should be noted that most situations of psychological vulnerability are the result of restrictive measures.

### 3.2. The consequences of the restrictive measures

The initial lockdown (introduced for the first time in Italy with the Ministerial Decree of 9 March 2020 “Io resto a casa”) is particularly relevant. Along with other subsequent restrictions such as the so-called red zones—Zones (cities or regions) in which the lockdown were active, on the basis of the monitoring of the disease trend—these measures led to significantly increased economic, social, and health vulnerabilities. Reductions in mobility, outdoor activities, closure of shops, meeting places and cultural venues are among the most impactful factors.

Many of the economic consequences such as reduced income, loss of work, but also difficulties with smart working are linked to these measures.

At the same time, the lockdown and restrictions have had wide-ranging consequences: from worsening lifestyles to the reduction of the access to care, hospitalization, treatment, monitoring of chronic diseases and prevention; from difficulties in managing distance learning, to children of families with greater social, economic and cultural difficulties dropping out of school.

Moreover, another large part of these consequences can be attributed to the increase in stress and the perhaps still difficult-to-measure consequences on the mental health of the population and again of the most fragile, the elderly and especially the young, often linked to the drastic cutting of social relations. An important example of this impact is the increase in eating disorders in Italy during the first 6 months of 2020, linked, among other things to the increase in exposure to social media (Vaccaro et al., 2021).

### 3.3. The intertwining of vulnerabilities

In addition, we also found an intertwining of vulnerabilities. We found several cases (43.7%) in which a subject fit into more than one category of vulnerability. Moreover, most participants (74.7%) had a pre-existing vulnerability. Some 38.5% of participants with (pre-existing?) vulnerabilities experienced an intensification of their vulnerabilities (Table 3).

**Table 3:** Compounded vulnerabilities (val.%).

	**Total interviews**	**New vulnerabilities due to COVID-19**	**Increased vulnerabilities due to COVID-19**
No initial vulnerabilities	25.3	54.5	0.0
Pre-existing vulnerability	74.7	63.1	38.5
One vulnerability	31.0	63.0	18.5
More than one	43.7	63.2	52.6
Total	100.0	60.9	28.7

The following examples show respondents whose vulnerabilities were compounded by COVID-19 (Table 4).

**Table 4:** The non-integrated foreign woman.

Woman	She studies medicine and works as a waitress, after going on layoff for COVID-19 she no longer has sufficient income and found a work in a call center
	40 years old
	Foreign
	Lives in Rome
She doesn't feel integrated in the place where she lives and she thinks that there are problems of integration that concern all foreigners toward whom there is a lack of interest from public institutions and also the associations of the third sector don't do enough	
**Vulnerability is a mixture of loneliness, uncertainty, anxiety, financial and health problems** “*I know people who are not doing well at the moment both economically and mentally. If one is sensitive or in difficult situations the body is affected. If a person is alone, has to support himself and does not have economic security, these elements affect the rest. And everything is increased by the anxiety of the moment*”	
**Living conditions and discrimination against foreigners create more forms of vulnerability** “*There are people of different nationalities and I think they are quite neglected. Their lifestyle and the environment they live in increases their health risks. My guess is that they don't even get tests for prevention and even forgo medical care for economic reasons of course. Those who work off the books would rather go to work even if they're sick than miss a day of work that won't be paid.”*	

In the second example, a woman with pre-existing health problems, which already had a major impact on her living situation, noted that becoming ill with COVID worsened her condition, also from an economic point of view. At the same time, finding a new job improved her psychological situation (Table 5).

**Table 5:** The woman with chronic diseases and long-COVID.

Woman	She lost her job because of COVID but recently found another one
	35 years old
	She lives alone
	She lives outside Rome
	She has chronic diseases and is obese, has long-COVID
Her illness had a major impact on her living situation (her boyfriend left her when he knew she was sick) but COVID also created psychological as well as physical problems to her	
She believes that a healthy environment is strategic to health, and likewise, good social relationships, which it is up to us to create	
**The psychological vulnerability related to COVID** “*The COVID had a strong emotional impact for me both physically and emotionally. I had to do two quarantines (40 days + 40 days). The fact that I can't get a hug or the fact that I can't invite people, to not see them physically at all has changed me so much.”* “*For months I felt that I had experienced a period of extreme loneliness.”*	
**Work as a tool for resilience** “*The biggest concern for me is having a stable job. Now that I have a job that I thought I couldn't do because of my physical problems, I feel more positive about facing life”*	

### 3.4. The impact on health

The impact on health is one of the most relevant aspects of the consequences of the pandemic in the strict sense and of the measures decided to combat its spread.

Some data from the first quantitative questionnaire testify this generalized health impact:

- 57% during the last 3 months was unwell (at least one day);
- 45% did not sleep well and among them, 27% because of COVID-related stress;
- 49% changed their (gained?) weight due to the COVID. The percentage rises to 61.2% among those who are overweight/obese;
- 42.1% of respondents spent most or most of their day sitting down.

The qualitative interviews highlighted many nuances of this impact on health, most notably psychological.

Considering COVID and its “disease” consequences, an increase in vulnerability is first and foremost evident, as can easily be expected, in people who have contracted COVID, but irrespective of the level of severity of the illness they have experienced, partly due to a lack of knowledge about the disease and a fear of being left more fragile in the face of future illnesses.

“*I had COVID about a year ago. But I still have ailments, especially a sense of asthenia, insomnia, I can no longer sustain work as I did before, both manual and concentration work. I feel old, I'm worried about how my body has reacted, I'm afraid of how it will react to other infections, even a simple flu. I try not to make the situation worse by avoiding harmful behaviours, such as smoking.”* ***(Male, 55-year-old, nurse, lives outside Rome)*. **

Caregivers experienced a worsening of their condition, even when their family members did not fall ill. On the one hand, there was increased concern that their parents or frail relatives they were caring for might become infected with SARS 2 and suffer serious consequences. On the other hand, because of the restrictive measures, they experienced loneliness and abandonment, without the support of other relatives or the same services.

“*By the way, my dad is immuno-depressed. So we had a lot of scruples many times. Today, fortunately, he's had his second dose of the vaccine and so we'll certainly be calmer for the future. Until now we've been really worried because if my dad had been infected it would have been the end of him because he's very, very weak.”* ***(Female, 46-year-old, housewife, caregiver, lives outside Rome)*. **

“*So this is the situation inside the house (two disabled parents), so imagine with COVID how I was obviously, we were extra careful, it was just absurd. that is, we all had to be careful because that applies to everyone, plus we really haven't done anything this year, nothing, always inside the house.”* ***(Female, 37 years old, secretary, caregiver, lives in Rome)*. **

Chronically ill patients also experienced a greater sense of health vulnerability, both because of the fear of falling seriously ill with COVID and because of the restrictive measures and fear of contagion themselves, which reduced their access to monitoring and treatment.

*I am worried because I have chronic bronchitis. In this situation I feel bad I even tried to commit suicide. From the beginning I had the impression that COVID was serious”* ***(Male, 63 years old, unemployed, has a citizenship income and a*** ***disability allowance. An earthquake victim, he lives between Abruzzo and Rome)*. **

“*At the moment I am not doing the checks I should because of the COVID, I preferred not to do it.”* ***(Female, 61 year old, with chronic illness, employed, lives in Rome)*. **

In addition to the distress created by the fear of illness, it should be noted that most situations of psychological vulnerability are the result of restrictive measures.

“*Because of the COVID by reducing sociality I am sadder, I struggle to do things, study, I sleep more.”* ***(Male, 29 years old, student, lives in Rome)*. **

“*This dark, tragic period has also stopped our emotionality, because we're scared, we're worried”* ***(Female, 43-year-old, on redundancy pay due to COVID, lives in Rome)*. **

Another negative effect on health, particularly widespread, is the decline in practices to sustain good health, linked to anxiety, fear of illness and restrictive measures introduced.

Very common is the experience of bad eating habits, much more carbohydrates and sweets, often homemade, also for their consoling and anti-stress power and the tendency to eat much more often during long hours at home. In fact, it is relevant to consider that the increase in the consumption of some types of food is linked with their symbolic value: the tendency to make bread, pizza and sweets at home, for example, it is linked to a specific food craving (especially carbohydrates) but could be interpreted as a pleasant way to spend time during a forced stay at home, and also as a way to deal with a difficult situation (Bracale and Vaccaro, 2020).

Reduced physical activity also had a great impact on health, both physical and psychological, and here too, the characteristics of the living environment made a difference. A larger house or one with outdoor space, the availability of parks or areas favorable to physical activity led to very different situations, with less impact on physical and mental health for those living in more facilitating contexts.

“*When there was lockdown we drank more alcohol, but not too much. There were times when we were quite nervous and maybe even at lunchtime we drank wine.”*

### 3.5. The great weight of economic vulnerabilities

But while the health vulnerabilities were important to our participants, they more frequently cited the disastrous economic consequences of the pandemic for them.

In this case, the preeminent role is certainly that of the restrictive measures that have blocked or reduced many economic and productive activities, especially in the tertiary sector, catering, tourism and many cultural and leisure activities (gyms, sports centers, cinemas, theaters, etc.).

The substantial impact is also evident from the quantitative questionnaire data:

- 48% of the VA sample experienced a decline? in income due to the COVID pandemic and, among those, 43% believed that it would not return to pre-pandemic levels;
- 73% of those experiencing a net decline in income as a result of the pandemic said their income was insufficient to sustain their desired standard of living.

Changes in the economic situation were linked to several aspects. Many respondents point to a major change from the previous situation. Economically strong groups such as restaurant entrepreneurs and shopkeepers in many categories were also affected.

These are often privileged categories, also from an economic point of view, who have experienced in a dramatic and unexpected way the consequences of a job characterized by entrepreneurial risk, as in the case of restaurateurs or shopkeepers who have had to close their businesses for long months, or by instability, such as that of freelancers or entertainment workers who have suddenly lost customers or spectators.

“*Until two years ago I was earning 2000–2500*€ *per month, and now for a reason, yes, they* explained it to me, but nobody cared that I had a job and I have nothing anymore.” ***(Male*,** ***55 years old, entertainment worker, lost his job due to COVID, lives in Rome)*. **

“*The pandemic has created new poor and therefore these new poverties are not covered.”* ***(Male, 56 years old, employee, lives in Rome)*. **

But alongside these new poor, for whom a recovery of their economic and income situation was likely when the restrictions come to an end, there is an awareness among the interviewees that the most serious consequences have affected those categories of workers who started from weaker positions in the labor market, those with precarious contracts or illegal workers, the youngest, women and immigrants. For these categories, the situation of greater economic vulnerability created by the measures to combat the pandemic, although mitigated by the new forms of welfare and subsidies introduced in the emergency phase, does not seem destined to change in a positive way over time.

“*For COVID, restaurant and pub owners and sports workers, who often do not even have a contract, are economically vulnerable.”*

“*A precarious person is economically vulnerable. He is someone like me. If I don't have 30 hours of lessons, if I only have 10, two people tell me from tomorrow that they don't have the money for the lessons and I can't buy food anymore. And maybe they are also prevented from doing their job, like me in the red zone.”* ***(Female, 35-year-old, personal trainer, lives*** ***outside Rome)*. **

“*If I didn't have help I wouldn't know what to do, in the end you have to pay the bills and have nothing left to eat. If I don't call friends to ask for food, I'm left with nothing. People I know, people I've done jobs for, who remain as acquaintances, kind people who tell me that if I call them they'll bring me something.”* ***(Transgender, 34 years old, gardener, lives outside*** ***Rome)*. **

### 3.6. Lesser-known vulnerabilities

The consequences of the pandemic are also reflected in other forms of vulnerability which appear less obvious, which have created discomfort often due to isolation and the new habits imposed by measures to contain the contagion, and which are often described as forms of anxiety, fear or disorientation. At the same time, participants allude to possible future consequences in relations between generations, in the face of the suffering of the elderly who have experienced a decisive rupture in relations with their children and grandchildren and who have spent the difficult times of lockdown or those of staying in RSA or possible hospitalization in solitude.

“*I had my grandfather who was hit by a stroke in hospital. And one thing I will never forget is the very nice nurse who handed the phone in face time to my grandfather... he was alone and in these cases a person could barely go. Support and never make people feel alone.”* ***(Female, 24-years old, university student lives in Rome)*. **

Moreover, it is interesting the reflection on the possible negative effects on the future development of the youngest, victims of the drastic cut in peer relationships. Not only adolescents, but also children, a silent section of the population on whom the effects of the pandemic in terms of increased vulnerability should be examined in depth. Many of them have experienced the situation of distance learning with difficulty, some have been cut off from it because of their family's initial difficulties, they have spent a lot of time alone in their rooms and on social networks, without being able to experience some important stages in their growth.

“*So vulnerable are the children and young people these days in my opinion. I saw my sister preparing for her baccalaureate at home. She did ‘the 100 days' at home. She didn't do the trip that classmates take together in their graduation year.”*

“*Or children with computers at 5 years old, and those at 8,9 years old who are happy to have the iPad so they can follow the lesson and they give them their homework on the iPad, does that seem normal? These are kids who haven't breathed a word but they exist. And it's important to keep an eye on them.”* ***(Female, 24-years old, university student, lives in Rome)*. **

Finally, our NVivo data analysis summarized the prevailing sentiments during the pandemic indicating the recurrence of certain sentiments.

**Figure 1:**
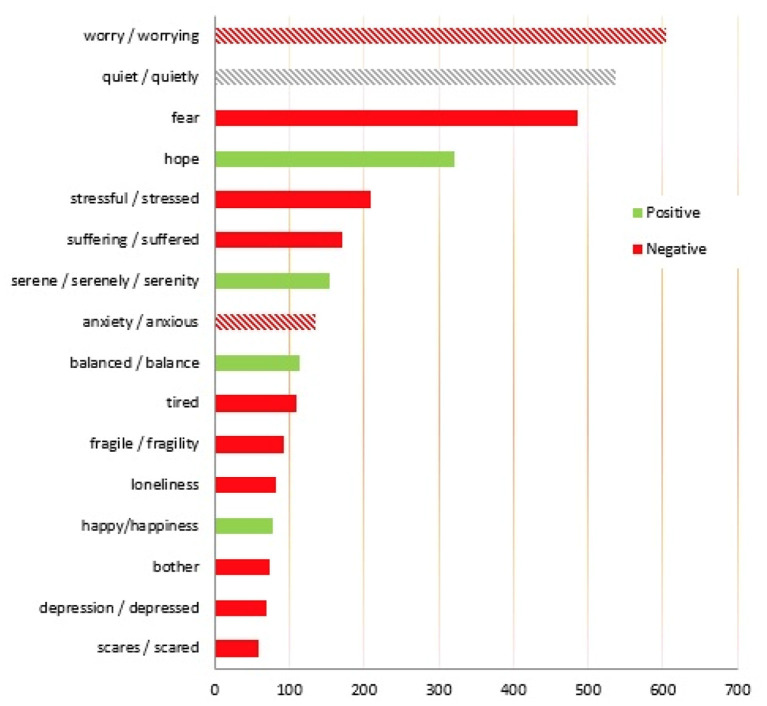
Words pronounced by the interviewee linked to moods and feelings (number of words > 50).

Fear prevails among all of them, although it was not present in the words used in the formulation of the questions. This state of mind, even if not necessarily explicit, emerges as a background reason from the tales of the interviewees and seems transversal to many conditions even if not marked by particular vulnerability (Figure 1).

When speaking of feelings of trust, it should be remembered that a minority of those interviewed expressed trust in the institutions, even before the emergency situation and independently of it, even if there are cases in which there has been an increase in this distrust precisely because of the management of the emergency.

“*I don't have much faith in institutions. Precisely because I think there is a lot of weakness and also in the government system... I don't feel very much... certainly the pandemic has made me change this view, you realise, as an entrepreneur, that if you have a problem nobody helps you. All they do is keep asking. We don't even have a trade union, to give you an example. The institutions don't give me confidence.”* ***(Male, 33 years old, entrepreneur, with a chronic disease, lives in Rome)*. **

There is also widespread criticism of the management of communication, which almost everyone describes as contradictory, redundant and confusing.

“*I believe that information is not that there is a lack of it, and that there is a lot of it and it is all different, so depending on the people you hear from, specialists, virologists or doctors in general, you hear a lot of different things that can create confusion and not reassure the person.”* ***(Transgender, 34 years old, gardener, lives outside Rome)***.

## 4. Discussion

The analysis has shown the complexity of the achieved concept of vulnerability on the ground experiences. First of all, vulnerabilities are often multiple and interconnected and can include several aspects: there is a biological/physiological vulnerability and an emotional/ psychological one. The socio-economic aspects are very relevant (e.g., isolated/ socially and/or financially disadvantaged) as well as the forms of cultural vulnerability. In any case, the results indicated that the health consequences of COVID (including psychological and mental health) tend to be more severe in the presence of existing health or economic vulnerabilities. Finally, it is interesting to point out that vulnerability to COVID itself can depend on individual behavior, strongly influenced by aspects such as trust in institutions, especially health institutions, and cultural aspects. More specifically, the level of information, on the one hand, and attitudes toward vaccinations, on the other, are two aspects that have greatly influenced behavior and also the vulnerability brought about by the pandemic experience. The set of results that describing the multidimensionality of the forms of vulnerability detected were also important because they formed the basis of the discussion during the Community Engagement phase and in the elaboration of action and intervention proposals.

## 5. Conclusion

The entire vulnerability/resilience assessment process is based on the fact that compounding multiple risk factors inhibit health agency, and eventually make it difficult or even impossible for people to become advocates for their own health destinies. In other words, the challenges of daily living for the most vulnerable can make it difficult if not impossible to capture their experience in other than ethnographic ways. This being so, nearly all survey techniques fail to reach the most vulnerable populations because these populations are so often unable to respond; for their loss of agency leaves them in a state of calamity coping in which the very thing that they are most at risk for cannot be attended to. We all know this; but figuring out how to study and characterize it as data has until now been a challenge, for individual ethnographies are episodic and hard to apply to larger populations. A recent publication called *The Economist* on the new *Health Inclusivity Index* (https://impact.economist.com/projects/health-inclusivity-index?i=2) describes the innovation of the SoNAR-Global research group in possessing proven methodologies for both identifying hidden vulnerable groups, but also for characterizing those otherwise invisible groups and bringing their lived experiences to the level of evidence—as already done for *Cities Changing Diabetes*. We also know this from an unpublished Vulnerability Assessment review study SoNAR-Global carried out for WHO, and from various reviews of Vulnerability Assessment strategies several of us co-published for SoNAR-Global and for Cities Changing Diabetes project: these procedures are broadly described in Napier and Volkmann (2023), *The Vulnerability Vortex: Health, Exclusion, and Social Responsibility*. The advantages of the emergence and empirical analysis of vulnerability are amplified by the possibility of their use in the shared elaboration of intervention proposals allowed by Community Engagement, which in the experience of the Italian study group has indeed yielded important results.

## Data availability statement

The datasets presented in this article are not readily available because the datasets used in the article are part of the SoNAR-Global European project and they're not transferable. Requests to access the datasets should be directed to https://www.sonar-global.eu.

## Ethics statement

The Ethics Committee of the Policlinico Umberto I of the University La Sapienza of Rome approved the Italian study with App. 6340 on 26/05/2021.

## Author contributions

Conceptualization: FL and CV. Methodology and supervision: DN, AV, and TG-V. Formal analysis and writing—original draft preparation: CV. Investigation: CV, DG, and GA. data curation: CV and GA. Writing—review and editing: FL. Visualization: CV. All authors have read and agreed to the published version of the manuscript.
